# Accuracy and practicability of a patient-specific guide using acetabular superolateral rim during THA in Crowe II/III DDH patients: a retrospective study

**DOI:** 10.1186/s13018-018-1029-1

**Published:** 2019-01-14

**Authors:** Chenggong Wang, Han Xiao, Weiwei Yang, Long Wang, Yihe Hu, Hua Liu, Da Zhong

**Affiliations:** 1 Department of Orthopedics, Xiangya Hospital, Central South University, No. 87 Xiangya Road, Changsha, 410008 Hunan China; 2 Department of Sports Medicine, Xiangya Hospital, Central South University, Changsha, Hunan China; 3 Geisel School of Medicine, Dartmouth College, Hanover, USA

**Keywords:** Total hip arthroplasty, Developmental dysplasia of the hip, Artificial acetabulum, Patient-specific instrument, Personalized operation

## Abstract

**Background:**

It is challenging to create an ideal artificial acetabulum during total hip arthroplasty (THA) in adult DDH. Our team developed a new patient-specific instrument (PSI) that uses the superolateral rim of the acetabulum as a positioning mark to assist in the production of an artificial acetabulum in adult Crowe II/III DDH patients. The purpose of this retrospective study is to verify whether this new PSI can be used to implement the preoperative plan accurately and quickly to create an ideal artificial acetabulum during THA in adult Crowe II/III DDH patients.

**Methods:**

We selected suitable adult Crowe II/III DDH patients from the registration system for artificial joint surgery at our hospital during April 2016 to March 2018 who underwent THA assisted by a PSI using the superolateral rim of the acetabulum as a positioning mark. We retrospectively analyzed data, including preoperative and postoperative anteversion, inclination, postoperative bilateral rotator center discrepancy (BRCD), surgery time, and the incidence of neurovascular injury. All patients underwent follow-up, and their Harris hip score (HHS) and X-ray data were recorded. Then, we performed statistical analyses on the data described above.

**Results:**

A total of 20 hip surgeries from 17 patients were included in our study. All patients underwent a successful operation assisted by the PSI. The mean anteversion of the cup in our preoperative plan was 15.1° (range, 10.0° to 20.0°), while the mean postoperative anteversion of the cup was 15.3° (range, 7.0° to 28.6°). The mean inclination of the cup in our preoperative plan was 44.7° (range, 40.0° to 50.0°), while the mean postoperative inclination of the cup was 45.6° (range, 35.0° to 57.6°). Paired-samples *t* test revealed no significant differences in anteversion and inclination between pre- and postoperation times (*P* > 0.05). The mean BRCD was 3.38 ± 3.0 mm (range, 0.5 to 11.0 mm). The average operation time was 105.1 ± 15.4 min, and no patients had neurovascular injury complications. All patients’ acetabular components appeared clinically and radiologically stable after surgery. The mean HHS values were significantly improved at 12 weeks (*P* < 0.05) and 24 weeks (*P* < 0.05) postoperatively compared to the preoperative mean scores.

**Conclusions:**

The new PSI is accurate and practical to create an ideal artificial acetabulum during THA in adult Crowe II/III DDH patients.

**Electronic supplementary material:**

The online version of this article (10.1186/s13018-018-1029-1) contains supplementary material, which is available to authorized users.

## Background

Total hip arthroplasty is a preferred treatment for patients with osteoarthritis secondary to developmental dysplasia of the hip (DDH) [1, 2]. For patients with Crowe type II/III DDH, acetabular abnormalities are always accompanied by malformations of relating structures, including a shallow acetabular fossa, a deficient acetabular wall, and contracted soft tissue, which makes it extremely difficult for surgeons to identify the real acetabulum and place the cup in a reasonable orientation [3, 4]. However, the cup position is key to the recovery of hip function and extends the lifespan of the implanted joint. Inaccurate placement of the cup can lead to severe postoperative complications, such as dislocation, impingement, worn prostheses, discrepancy of the low limbs, and a high rate of revision [5–7]. The conventional method highly relies on the skill and prior experience of the surgeon who performs the operation and can be inconsistent and unsatisfactory [8–11].

With the development of CT and rapid prototyping technology (RPT), we can easily reconstruct a digitized pelvis and generate an equal scale pelvis model to simulate the placement of the cup [12–15]. This method is beneficial for the design of high-quality and personalized operation plans, but completely conducting the plan in a real operation is not easy given situational differences and factor variability. Is it possible to realistically and accurately perform the operation plan?

In the past, many groups have attempted to address this problem. Meermans G et al. [16] found that the transverse acetabular ligament but not acetabular component inclination may be used to obtain the appropriate anteversion when introducing the acetabular component during total hip arthroplasty (THA). Epstein NJ et al. [17] found that the transverse acetabular ligament could not be routinely identified at surgery, but this method was not more accurate for cup positioning compared with the free-hand technique. Hiroyuki Ogawa et al [18] designed an AR-HIP system to assist the surgeon in judging the orientation during the operation, but the clinical feasibility remains unknown. Chen B et al. [19] created a plate to guide the reaming acetabulum. However, the interruption of soft tissues cannot be avoided; thus, the clinical use is limited. Currently, navigation technology is perhaps one of the most pervasive methods for the accurate placement of the cup. Using this technology, the surgeons are able to determine the real position of the acetabulum and place the cup in an ideal orientation. However, the high cost and increased complexity of the procedure largely restrict its application. At the same time, there are some studies that reported no improvement in accuracy and no benefit between the traditional method and navigation technology [20–22]. Therefore, a new method that could facilitate the accurate location of the real acetabulum and the perfect orientation of the cup in Crowe type II/III DDH patients is needed.

To solve this problem, we designed a patient-specific instrument (PSI) based on three-dimensional (3D) reconstruction and RPT technology that uses the superolateral rim of the acetabulum as a positioning mark to assist in the production of an artificial acetabulum in adult Crowe II/III DDH patients. The technology, which has been validated by numerous model trials and approved by ethics committees, was employed in a clinical study in our hospital. Therefore, we selected qualified cases from the artificial joint surgery registration system of our hospital for this retrospective study. The purpose of this study is to verify whether this new PSI can carry out the preoperative plan accurately and quickly and to create an ideal artificial acetabulum during THA for adult Crowe II/III DDH patients.

## Methods

### Retrospective study design

This retrospective study was conducted according to Declaration of Helsinki principles and was approved by the medical ethics committee of the Xiangya Hospital of central South University. We retrospectively analyzed Crowe type II/III patients who underwent THA during April 2016 to March 2018 in our hospital. We selected suitable patients who underwent the same treatment during the perioperative and postoperative follow-up from the registration system for artificial joint surgery in our hospital. The inclusion criteria were as follows: (1) primary THA, (2) the quality of bone was sufficient to place the cup in the true acetabulum, (3) surgery without osteotomy of the trochanteric, and (4) surgery with the guide of the instrument. The patients we selected had the same preoperative planning, surgery implementation, evaluations, and measurements.

### Patients

By carefully searching the registration system for artificial joint surgery in accordance with our study design, 17 patients were enrolled in our study. The subjects included 5 men and 12 females with an average age of 50.35 ± 15.74 years (range 22 to 77 years old). All patients were diagnosed with Crowe type II/III DDH by an experienced orthopedic surgeon through standard pelvis radiography. Nine patients were diagnosed with Crowe type II, and 11 were diagnosed with Crowe type III. Basic information of the patients is presented in Table 1.

**Table 1:** Demographic characteristics of the patients with Crowe types II and III DDH

Patient	Gender	Age (years)	Sides	Crowe classification
1	F	51	Bilateral	II (Left)/II (Right)
2	F	54	Bilateral	III (Left)/III (Right)
3	F	26	R	III
4	M	63	L	III
5	F	66	R	II
6	F	22	L	II
7	F	32	L	III
8	M	30	R	II
9	F	53	L	III
10	M	49	L	III
11	F	50	R	II
12	F	63	L	III
13	F	77	L	II
14	M	44	R	II
15	F	48	Bilateral	III (Left)/ II (Right)
16	F	71	L	III
17	M	57	L	III
Sum or mean	5 M/12F	50.35 ± 15.74	8R/12 L	9 II/11 III

### Preoperative planning

#### Reconstruction of the pelvis and simulation of prosthetic implantation

CT scanning data of the pelvis from patients were exported for preprocessing from a Philips scanner (Philips, Eindhoven, Netherlands) with 0.6-mm slice thickness and were saved in a DICOM format. Briefly, 3D models of the pelvis were digitally reconstructed using Mimics 19.0 software (Materialize, Leuven, Belgium). We first determined the coronal plane based on the relative position of the anterior superior iliac spines and the pubic tubercles. Then, the pelvis position was standardized with reference to the anterior pelvic plane [3].

**Fig. 1:**
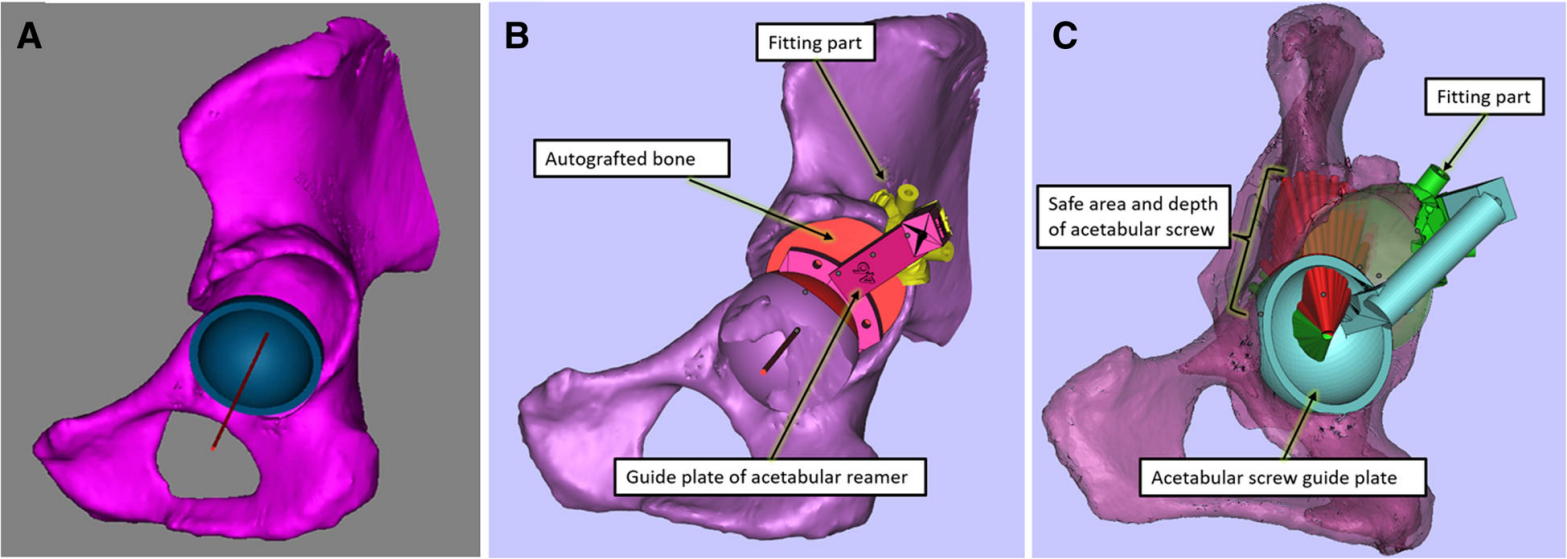
**a** We simulated the implantation of the cup to obtain the ideal cup position. **b** The fitting part of the guide plate (yellow-green part) and guide plate of the acetabular reamer (rose red part) were designed. **c** The acetabular screw guide plate includes the hollowed-out area that perfectly corresponds to the safe penetration area of the acetabular screw. The red cylinder presents the relative safe penetration area of the acetabular screw through the graft bone zone. The green cylinder indicates the absolute penetration area of the acetabular screw

We mimicked the implantation of the cup in the real acetabulum according to the anatomic characteristics of the patient’s acetabulum. The 3D, sagittal, coronal, and transverse views were presented to determine the ideal position based on the following criteria: (1) the diameter of the cup was confined by the peripheral border of the real acetabulum to achieve the so-called rim fit; (2) the cup size was chosen to best accommodate the anteroposterior of the real acetabulum; if the contralateral head was normal, we could duplicate its size and rotator center position to the affected side; and (3) the cup exhibited good bone coverage; in general, we designed the coverage to be greater than 70% (Fig. 1a). Of note, although the acetabulum was stable according to our preoperative design, we used a structural bone graft during the surgery when the acetabular defect was large to provide more bone mass for the next revision surgery, considering that DDH-THA patients are generally young.

#### Preoperative design of the PSI

Once we determined the ideal cup size and position, we developed a PSI to replicate the position of the implantation during surgery. This instrument consisted of three parts (Fig. 1b, c):

- The fitting part: we chose part of the superolateral rim of the acetabulum and analyzed the surface to design a fitting part that matched the unique bony landmarks.
- The guide plate of the acetabular reamer: this part included one end connecting to the fitting part and one arc-shaped end matching the simulated cup rim surface. This special part can help guide the reaming size, orientation, depth, and placement of the cup.
- The acetabular screw guide plate: given that the axis of the tack hole passed through the rotator center, we mimicked the possible orientation of the screw and removed the screws outside the acetabular bone. The projection area of the screw on the cup was within the safe zone. The acetabular screw guide template was produced such that the hollow-out area perfectly corresponded to the safe zone of the acetabular screw. The length of the acetabular screw was also simultaneously obtained according to the safety depth.

**Fig. 2:**
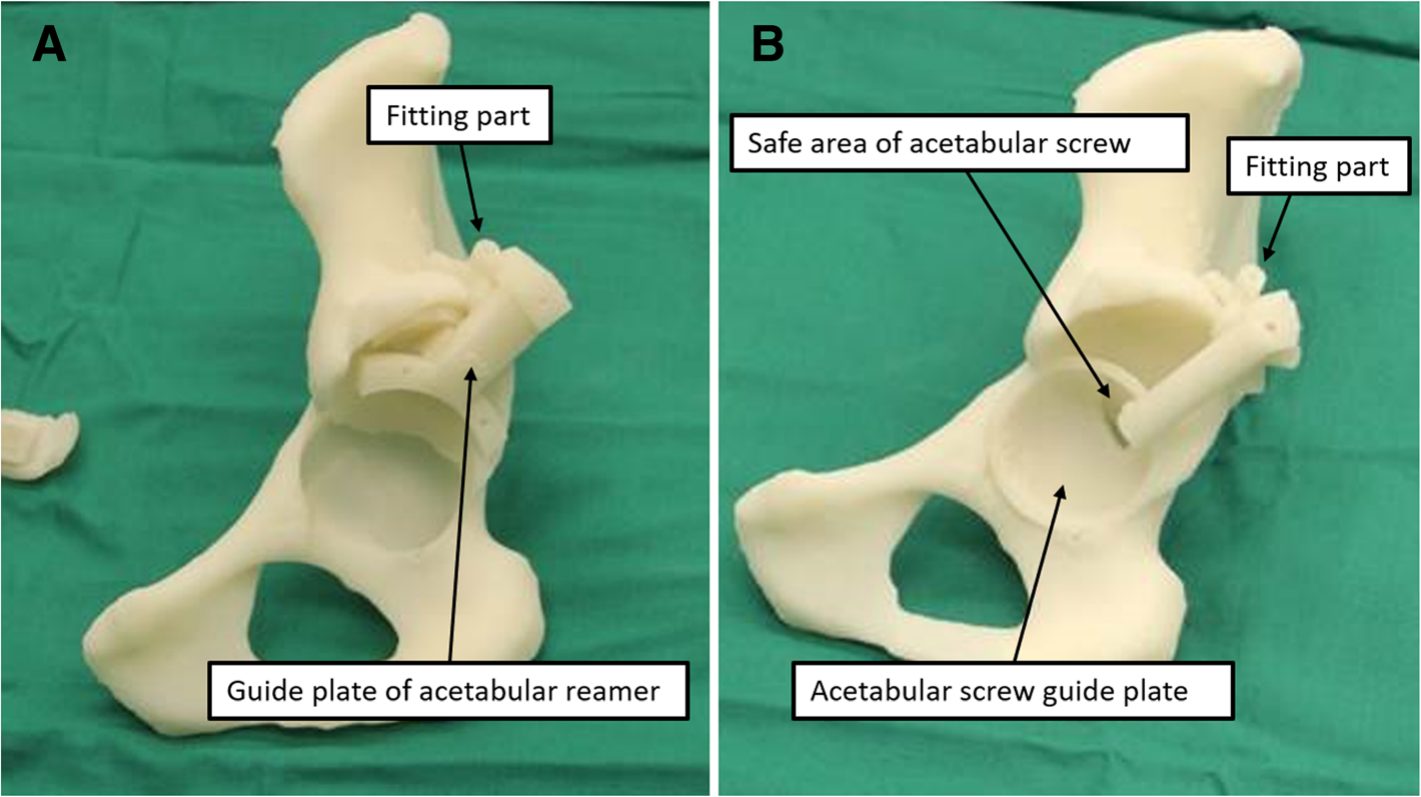
Inspection of the guide plate. **a** The fitting part and guide plate of the acetabular reamer were assembled on the acetabular model. **b** The fitting part and guide plate of acetabular screw were assembled on the acetabular model


Finally, a PSI was generated based on the anatomical structure of the acetabular contour and tube. We used nylon material to make the instrument and pelvis using the selective laser sintering (SLS) technique. Only qualified instruments were sterilized and used in the surgery after strict inspection (Fig. 2). An example of the designing process is presented in Additional file 1: Video S1.

Additional file 1:**Video S1.** An example of the PSI preoperative design process. (MP4 27671 kb)


### Surgery

**Fig. 3:**
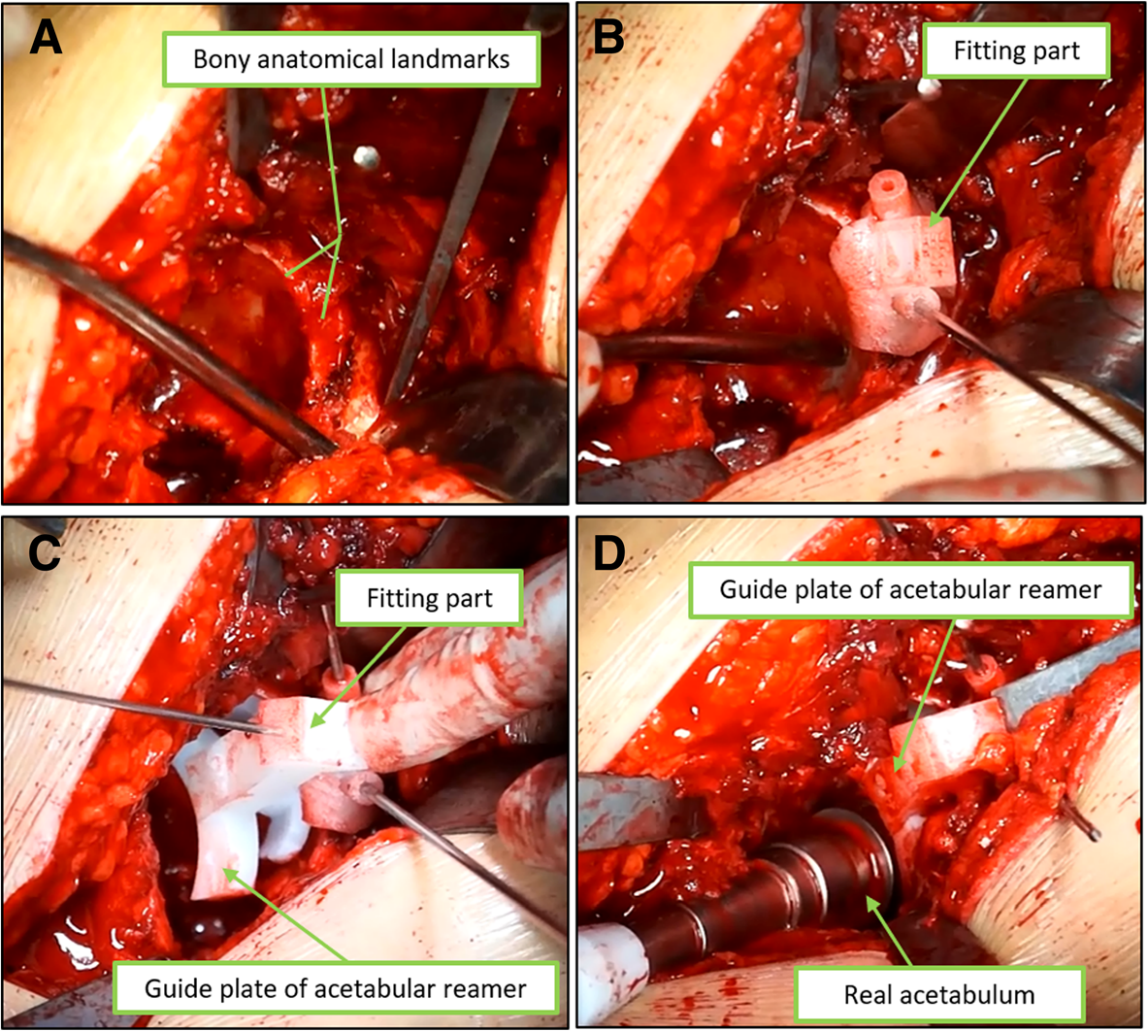
**a** Exposure of the superolateral rim to the acetabulum. **b** To match the fitting part into the acetabulum, 3 K-wires were placed and fixed to the bone through the plate hole. **c** The guide plate of acetabular reamer was placed to the fitting part. **d** The acetabulum was reamed under the guidance of the plate of acetabular reamer


The surgeon who participated in the design of the PSI performed all operations via a direct posterior-lateral approach. We dislocated the hip and dissected the head. Then, we followed the steps to use the instrument. First, the superolateral portion of the acetabulum was fully exposed, and the fitting part, which was similar to a lamp, was inserted into the unique suitable place, and three K-wires with an appropriate diameter were placed and installed in the bone through the plate hole. Second, the acetabular reamer instrument was placed and fixed to the fitting part. We used and followed the acetabular reamer instrument to drill and shape the prosthetic shell (Fig. 3). If the acetabulum defect was severe, we used a structural bone graft during surgery to provide more bone mass for the next revision surgery. For patients with Crowe II or mild acetabular defects in the real acetabulum area, we simply grafted the bone after crushing the femoral head. Third, we often use additional acetabular screws to reinforce the acetabular cup; thus, the acetabular screw guide device was installed to the fitting part. We marked the hollowed-out area with methylthionine. The acetabular cup was inserted with the screw hole within the methylthionine-marked area. Then, we pressed the cup and inserted the screws. The implantation of the inner diameter ceramic liner was performed as the last step. An example of the surgical process is presented in Additional file 2: Video S2.

Additional file 2:**Video S2.** An example of the surgery process using PSI. (MP4 159510 kb)


### Evaluations and measurements

**Fig. 4:**
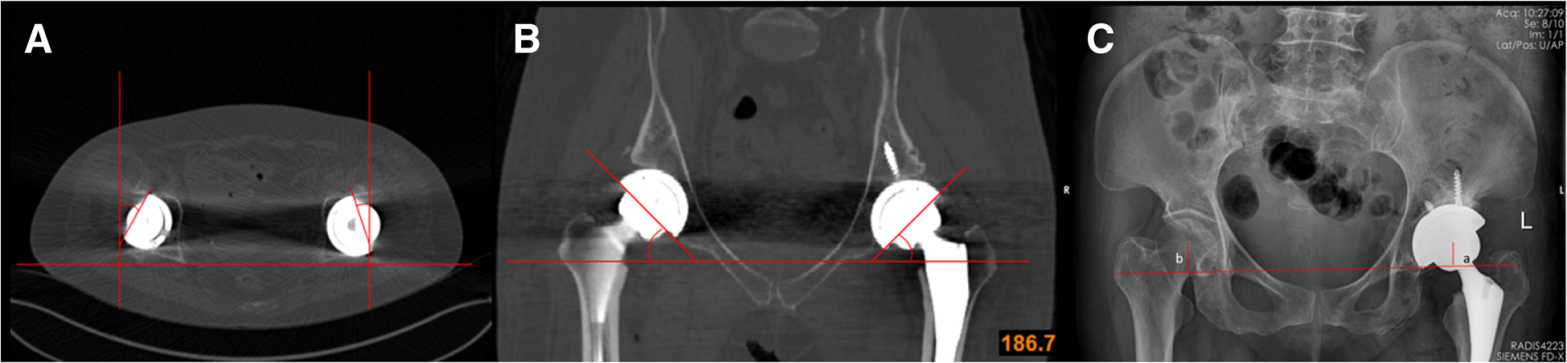
We used computed tomography to present the transverse view to measure the anteversion (**a**) and the coronal view (**b**) to measure the inclination. We used a standard pelvis anterior-posterior X-ray (**c**) to assess the BRCD (BRCD = |b-a|). a and b represent the distance between the rotator center and the line that passes through the bilateral teardrop

We collected the preoperative Harris hip scores (HHS) [23] of patients. We also collected the preoperative ideal anteversion and inclination data and performed standard radiological examinations (X-rays, CT) postoperatively for all patients. Cup abduction, anteversion, and bilateral rotator center discrepancy (BRCD) were measured from these examinations (Fig. 4). The surgical time per hip as well as the blood and nerve injury complication rates were also calculated and evaluated during or after surgery. All patients underwent follow-up. HHS was recorded 12 and 24 weeks after surgery, and X-rays were obtained 12 weeks after surgery.

### Statistical analysis

Data are presented as means with ranges. Paired-samples *t* test was used to analyze the pre- and postoperative abduction angle, pre- and postoperative anteversion angle, deviation of pre- and postoperative cup anteversion and inclination, and differences in pre- and postoperative HHS. We adopted the Bland-Altman plot to evaluate the agreement between planning and actual cup position. Statistical analyses were performed using SPSS 22.0 software (SPSS Inc., Chicago, IL, USA). *P* values less than 0.05 were considered statistically significant.

## Results

**Fig. 5:**
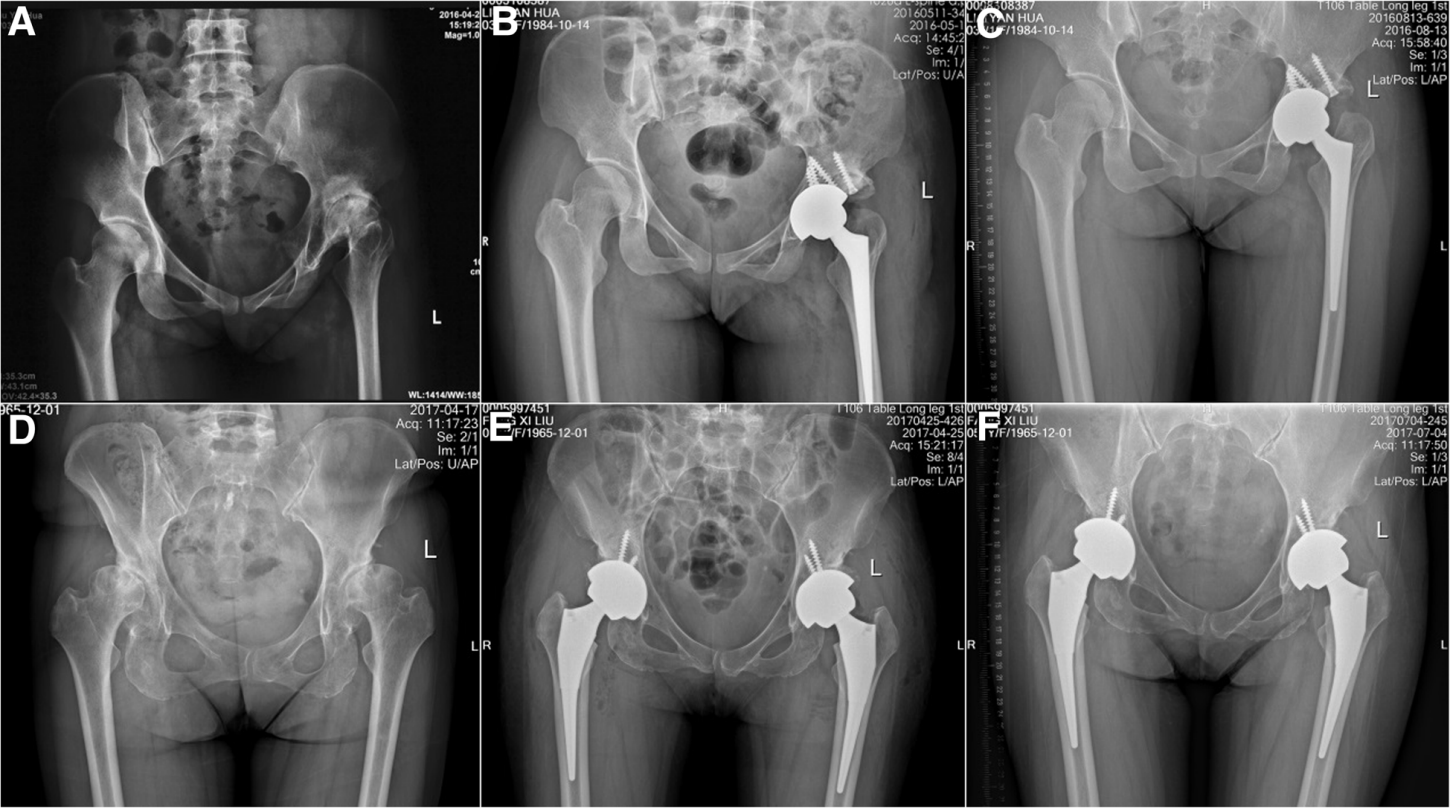
Samples from our patients. **a** The preoperative X-ray of patient 1: a 32-year-old female diagnosed with the Crow type III DDH of the left hip. **b** The postoperative X-ray of patient 1 (2 days after surgery): the designed inclination was 45° (left), whereas the anteversion was 15° (left). The postoperative inclination was 48.3° (left), whereas the anteversion was 18.4° (left). **c** The postoperative X-ray of patient 1 (3 months after surgery): the acetabular components appeared radiologically stable. **d** The preoperative X-ray of patient 2: a 51-year-old female diagnosed with the Crow type II DDH on both sides of the hips. **e** The postoperative X-ray of patient 2 (4 days after surgery): the designed inclination values were 46° (left) and 45° (right), whereas the anteversion values were 10° (left) and 18° (right). The postoperative inclination values were 48.7° (left) and 41.8° (right), whereas the anteversion values were 7° (left) and 21.6° (right). **f** The postoperative X-ray of patient 2 (3 months after surgery): the acetabular components appeared radiologically stable

All the surgeries were performed by one surgeon with the help of the instrument. Three patients received simultaneous bilateral THA. Fourteen patients received unilateral THA (Fig. 5). Three patients accepted structural bone grafts fixed with two screws. In one operation, the cup was fixed with three screws, and the cup was fixed with two screws in sixteen operations. In the remaining three operations, the cup was fixed with one screw. The duration of the surgeries for unilateral THAs was 105.1 ± 15.4 min (range, 85 to 142 min). No neurovascular complications were noted within 1 week after the surgery (Table 2).

**Table 2:** The information of the surgeries

Case	Sides	Structural bone graft	Blood and nerve complications	Surgery time (min)
1	Bilateral	N	N	90/94
2	Bilateral	N	N	96/95
3	R	N	N	100
4	L	N	N	120
5	R	N	N	95
6	L	N	N	105
7	L	Y	N	138
8	R	Y	N	142
9	L	N	N	118
10	L	N	N	108
11	R	N	N	95
12	L	N	N	103
13	L	N	N	90
14	R	N	N	100
15	Bilateral	N	N	85/102
16	L	N	N	105
17	L	Y	N	121
Sum or mean	8R/12 L	3Y/14 N	0Y/17 N	105.1 ± 15.4

The mean anteversion of the cup in our preoperative plan was 15.1° (range, 10.0° to 20.0°), while the mean postoperative anteversion of the cup was 15.3° (range, 7.0° to 28.6°). These values were comparable without statistically significant differences (paired-samples *t* test, *P* = 0.736). The mean inclination of the cup in our preoperative plan was 44.7° (range, 40.0° to 50.0°), while the mean postoperative inclination of the cup was 45.6° (range, 35.0° to 57.6°). No significant difference was identified between these parameters (paired-samples *t* test, *P* = 0.379). The mean BRCD was 3.38 ± 3.0 mm (range, 0.5 to 11.0 mm). The deviation of anteversion was 2.7 ± 2.0° (range, 0.4° to 8.6°), while the deviation of inclination was 4.2 ± 2.5° (range, 1.1° to 10.1°) (Table 3).

**Table 3:** Data of preoperative plan and postoperative measurement and related comparison

Case	Inclination (unilateral or L/R)			Anteversion (unilateral or L/R)			BRCD (mm)
	Preoperative plan (°)	Postoperative (°)	Δ (°)	Preoperative plan (°)	Postoperative (°)	Δ (°)	
1	46.0/45.0	48.7/41.8	2.7/3.2	10.0/18.0	7.0/21.6	3.0/3.6	1
2	45.0/47.0	46.1/ 57.1	1.1/10.1	15.0/20.0	17.8/28.6	2.8/8.6	7
3	40.0	35.0	5.0	13.0	15.0	2.0	0.5
4	44.0	37.2	6.8	15.0	15.4	0.4	4
5	45.0	47.3	2.3	15.0	14.4	0.6	3
6	40.0	43.3	3.3	12.0	9.2	2.8	8.7
7	45.0	48.3	3.3	15.0	18.4	3.4	5
8	45.0	46.5	1.5	13.0	9.4	3.6	2
9	50.0	57.6	7.6	15.0	15.8	0.8	2
10	45.0	49.7	4.7	17.0	20.1	3.1	11
11	42.0	37.5	4.5	15.0	14.1	0.9	2
12	42.0	50.1	8.1	15.0	16.6	1.6	4.2
13	45.0	47.5	2.5	15.0	15.5	0.5	0.9
14	46.0	39.6	6.4	17.0	14.2	2.8	2
15	46.0/45.0	42.2/48.0	3.8/3.0	17.0/13.0	12.1/15.3	4.9/2.3	1.2
16	45.0	46.2	1.2	16.0	11.6	4.4	1.8
17	45.0	42.8	2.2	15.0	14.0	1.0	1.1
Mean	44.7 ± 2.3	45.6 ± 5.9	4.2 ± 2.5	15.1 ± 2.2	15.3 ± 4.7	2.7 ± 2.0	3.38 ± 3.0
Paired-samples *t* test	*t* = − 0.901, *P* = 0.379		/	*t* = −0.342, *P* = 0.736		/	/

**Fig. 6:**
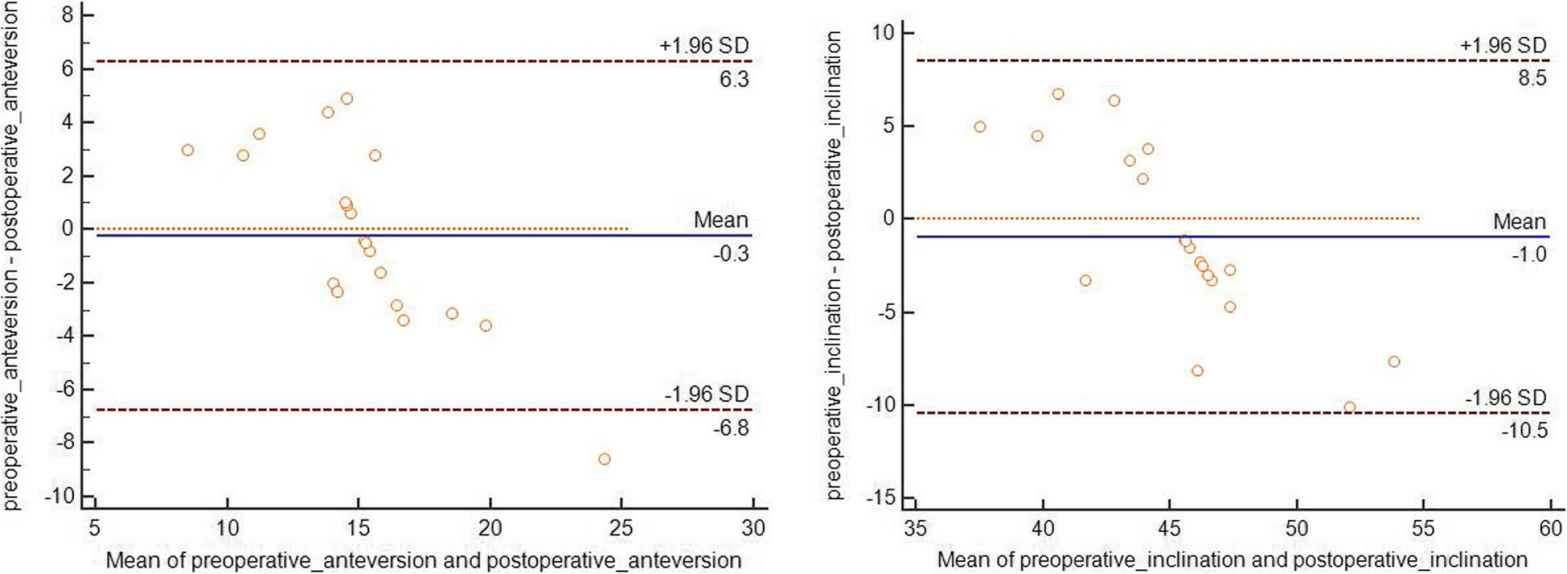
Bland-Altman analysis of the anteversion and inclination. The difference represents the difference between the preoperative anteversion and postoperative anteversion as well as the preoperative inclination and postoperative inclination. The blue line represents the mean bias. The dashed red lines represent the 95% limits of agreement. *SD* standard deviation

**Fig. 7:**
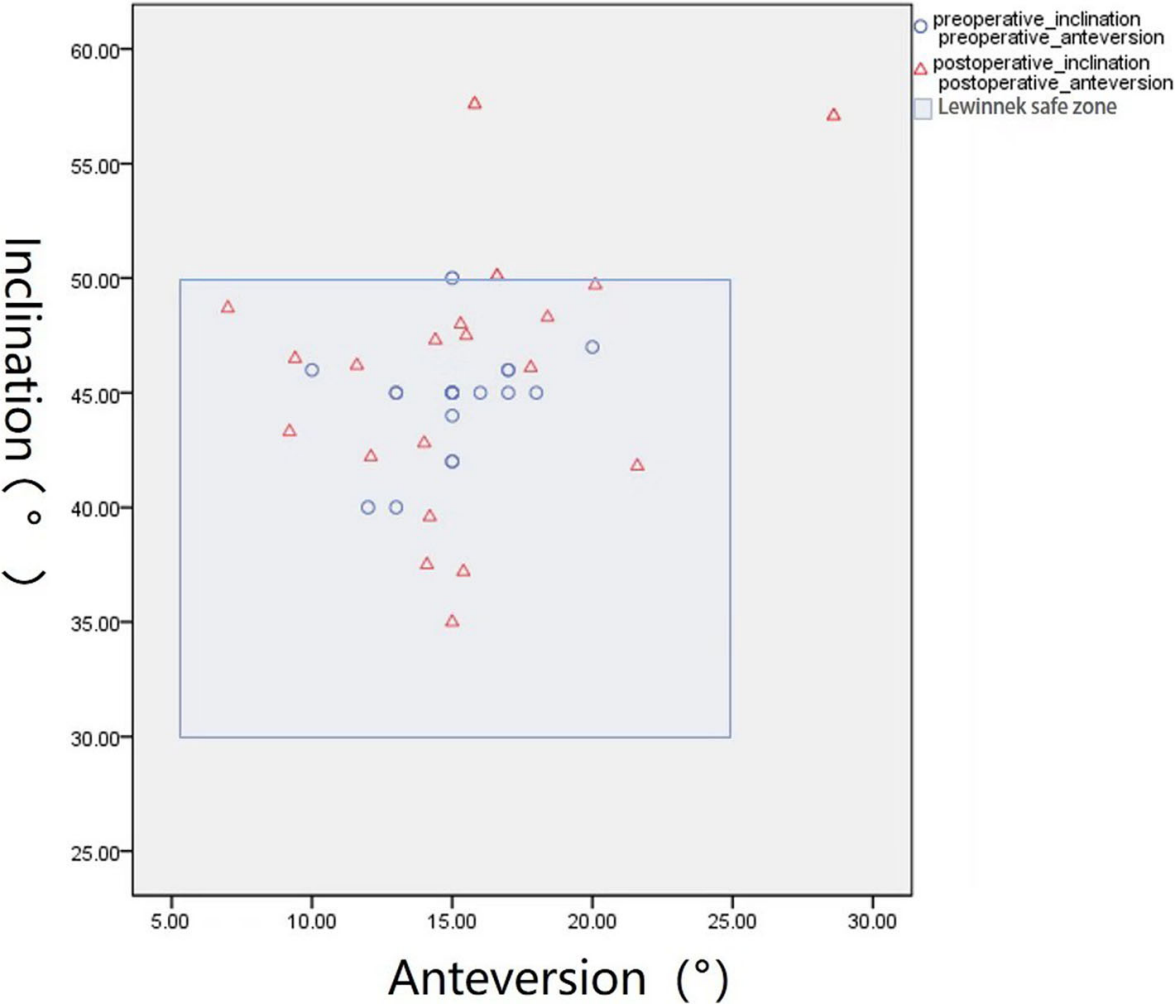
The scatterplot shows the position of the cup. The green plots represent the preoperative cup position. The blue plots represent the postoperative cup position. The frame represents the Lewinnek safe zone

Bland-Altman analysis revealed good agreement between the postoperative and preoperative cup position (Fig. 6). According to the Lewinnek safe zone definition [24], two hips were located outside the safe zone; thus, the percentage of outliers was 10% (Fig. 7).

Sixteen patients were followed up twice, and 1 unilateral patient only completed the follow-up at 12 weeks after surgery. All patients’ acetabular components appeared clinically and radiologically stable after surgery (Fig. 5). The HHS at 12 and 24 weeks after surgery was increased compared with that before surgery (*P* < 0.05), and the HHS at 24 weeks after surgery was increased compared with that at 12 weeks after surgery (*P* < 0.05) (Table 4).

**Table 4:** The changes and paired samples *t* test of pre- and postoperative HHS

	*n*	HHS mean	*t* value	*P* value
Preoperative	17	40.65 ± 9.66	− 20.518	0.000
Postoperative (12 weeks)		87.29 ± 4.40		
Preoperative	16^a^	39.94 ± 9.51	− 22.796	0.000
Postoperative (24 weeks)		92.38 ± 2.03		
Postoperative (12 weeks)	16^a^	87.25 ± 4.54	− 5.149	0.000
Postoperative (24 weeks)		92.38 ± 2.03		

^a^One patient only completed the follow-up at 12 weeks after surgery

## Discussion

This is a retrospective study that seeks to verify whether the PSI using the acetabular superolateral rim as a positioning mark can carry out the preoperative plan accurately and quickly to create an ideal artificial acetabulum during THA in adult Crowe II/III DDH patients. Therefore, we discuss this study from three aspects: the design of the PSI, the application of the PSI, and the limitations of the PSI and study.

### The design of the PSI

This study is not the first report of a PSI applied in THA [25], but the PSI developed by our team is original and new. There are three main substantial differences of our new PSI compared with other PSIs: positioning mark, raw materials and preparation technology, and surgical indications.

First, regarding the selection of the PSI positioning mark, some PSI positioning marks are designed for the acetabular fossa [26], which will cause large errors. The acetabular fossa contains a large amount of soft tissue. CT data can only recognize osseous boundaries because it is very difficult to remove the soft tissue in the acetabular fossa to reveal the real osseous boundaries completely. Thus, it is possible that the PSI will not fit stably in the positioning mark. As a result, large errors will occur. Based on numerous model tests, computer simulations, and clinical studies, we believe that the use of the superolateral rim of the acetabulum as a positioning mark is an ideal choice for acetabular PSI. Given the limited soft tissue coverage of the superolateral rim of the acetabulum, it is easy to reveal the real osseous boundaries completely.

Second, we discuss the raw materials and preparation technology. Currently, the production technology of other THA-PSI systems involved fused deposition modeling (FDM) or stereo lithography appearance (SLA) [25, 26], but our new PSI uses selected laser sintering (SLS) technology. The disadvantages of FDM include the low melting point of FDM products and soft texture [27]; these features will render FDM products unusable with high-temperature disinfection and will easily cause FDM product deformation during the operation. One disadvantage of SLA is that the SLA product texture becomes brittle after disinfection, and relevant reports suggest that SLA products exhibit a certain toxicity [28]. Our new SLS-PSI that uses nylon as the raw material is a nontoxic, high-temperature sterilization, and non-deformation product that represents a more ideal choice for acetabular PSI.

Third, we discuss surgical indications. Combined with a large number of model tests, computer simulation surgeries, clinical studies, and relevant literature [29], we considered that the PSI is not required in THA for adult Crowe I DDH patients. Thus, we focused on the application of the PSI in THA for adult Crowe II/III DDH patients. In addition, although we also developed the THA-PSI for adult Crowe IV DDH patients, the design principles and surgical methods for our PSI significantly differ from those of Crowe II/III. The positioning mark of THA-PSI for adult Crowe IV DDH patients does not use the superolateral rim of the acetabulum; the false acetabular fossa is used. Therefore, the most suitable surgical application for the new PSI is to guide the production of the acetabulum in adult Crowe II/III THA patients.

### The application of PSI

In this paper, our results showed that the PSI had good clinical efficacy in the treatment of Crowe II/III patients. We will discuss the advantages of the instrument based on the following 4 aspects.

First, the guide plate helps to quickly identify the true acetabulum. Previous study claims that, although it is technically easier to locate the prosthesis in a high rotator center for high hip dislocation, the real acetabulum remains the best position for the prosthesis in Crowe II/III DDH patients [30]. Typically, the first step to locate the real acetabulum is to identify the cotyloid fossa and the inferior aspect of the teardrop; however, uncertainties among different anatomical regions increase the difficulty of the surgery. Surgeons who perform the surgery must be very experienced to handle such situations to avoid bleeding and nerve injury in the patient. Taking this into consideration, we decided to first locate the superolateral rim of the acetabulum, which is easily exposed through the posterior-lateral approach. The clamp-like fitting part can only match the unique rim with a particular angle, similar to how a key matches a unique lock; therefore, there is a reduced risk for inaccurate placement or other mistakes. Fixing three K-wires through the fitting part enhances the stability during surgery to avoid the influence of the patients’ movement. Before dissecting the soft tissue, we can clearly identify the real acetabulum with the help of the guide plate of the acetabular reamer, which reduces the surgery time and decreases the rate of bleeding and nerve injury.

Second, we can objectively guide acetabular grinding. Bony deficiency is another troublesome issue that surgeons often face during reconstruction of the acetabulum. Complete preoperative planning is needed to ensure adequate bone coverage while avoiding the over reaming of the acetabulum [31, 32]. Our PSI restricts the maximum size and depth of the reamer to promote close matching of the acetabulum to avoid over reaming. When a structural bone graft is needed, we can easily implant the truncated femoral head with the help of the acetabular reamer guide plate to restrict its shape. In our study, all the cup sizes are consistent with preoperative planning. In two cases, patients received a structural bone graft along with THA. Both cases revealed profound initial stability after surgery. No sign of over reaming was noted in either case.

Third, we can accurately replicate the cup orientation under the guide of the plate. To ensure the accuracy of placing the cup in a suitable position during operation, we always used the acetabular component alignment guides and the transverse alignment as a reference. Grammatopoulos G et al. [33] demonstrated that the use of visual cues helps to enhance accuracy, while conventional techniques result in a large variability in acetabular component orientation. New and better guides and methods for training should be developed. Minoda Y et al. [10] suggested that the usage of modern alignment guides inherently mislead anteversion to a mean reduction of 6° (maximum, 12°) and inclination to a mean increase of 2° (maximum, 4°). Such alignment guide settings could be sources of error in acetabular component orientation. Our PSI could guide the surgeon to place the cup according to plan; therefore, even surgeons with less experience can successfully and accurately decide the orientation. The high consistency of the position between our samples supports this notion.

Fourth, the PSI can safely guide the placement of the screw. For DDH patients, the safe zone for the fixation of the acetabular screw should be thoroughly investigated to avoid neurovascular injury. Wasielewski et al. [34] developed a quadrant system to define a safe fixation zone in the normal acetabulum. Liu Q et al. [35] used 3D technology to create a new safe zone to guide the screw fixation for high dislocation DDH patients. However, this information only provided a theoretical understanding of where to place the screw. In real operation, the condition is highly variable among different patients, and surgeons still have trouble determining the ideal position for placing the screw. Our acetabular screw guide plate is specifically designed to address this problem. We can directly mark the safe zone on the acetabulum during the operation. The hole depth was acquired preoperatively so that the rate of complications during surgery is extremely low.

### Limitations

The PSI in this study has several limitations. First, some technical challenges need to be overcome. A three-dimensional printing device is required, and the skilled ability of the three-dimensional design technology is mandated. Second, standardization of the design process without universal software may limit the promotion and application of the three-dimensional patient-specific acetabular bony landmark navigational technique in total hip arthroplasty. Third, this is a preliminary application with a small sample size and a short follow-up period. Therefore, further prospective investigations with larger sample sizes and longer follow-up durations are necessary to investigate appropriate values for clinical application.

## Conclusions

The new PSI uses the superolateral rim of the acetabulum as a positioning mark can indeed carry out the preoperative plan accurately and quickly. Thus, we believe that the new PSI is accurate and practical to create an ideal artificial acetabulum during THA in adult Crowe II/III DDH patients.

## Abbreviations

BRCD: Bilateral rotator center discrepancy
DDH: Developmental dysplasia of the hip
FDM: Fused deposition modeling
PSI: Patient-specific instrument
RPT: Rapid prototyping technology
SLA: Stereo lithography appearance
SLS: Selected laser sintering
THA: Total hip arthroplasty
